# Wildfire as a natural stressor and its effect on female phenotype and ornament development

**DOI:** 10.1002/ece3.7457

**Published:** 2021-03-23

**Authors:** Stacey L. Weiss, Robert M. Brower

**Affiliations:** ^1^ Department of Biology University of Puget Sound Tacoma WA USA

**Keywords:** condition‐dependent signals, corticosterone, fire ecology, herpetofauna, prescribed fire, *Sceloporus*, sexual selection, stress

## Abstract

Controlled low‐intensity fires are commonly used in ecosystem management for both habitat restoration and wildfire management. Animals in those ecosystems may respond to fire by shifting energy allocation away from reproduction and growth, and toward maintenance. Stress‐induced shifts in energy allocation may affect the expression of condition‐dependent sexual signals, which are sensitive to energetic and physiological trade‐offs mediated by glucocorticoids. Here, we examine the effect of fire on ornament expression, corticosterone, and other phenotypic traits in a population of striped plateau lizards, *Sceloporus virgatus*, affected by the Horseshoe 2 Fire in the Chiricahua Mountains, Arizona, USA. The condition‐dependent female ornament was significantly smaller the month following the fire than 2 years prior and was both smaller and less orange on the burned site relative to a nearby unburned site. These patterns are similar to those found in a previous experimental study examining the response of the ornament to corticosterone manipulations. Yet, in the current study, corticosterone levels were not different in lizards on the burned and unburned sites. Perhaps glucocorticoid levels already returned to baseline, or do not adequately track environmental change. Females tended to be smaller and lighter on the burned site than the unburned site; however, the year after the fire, body condition was higher for females on the burned site, indicating a rapid recovery and potential long‐term benefits in response to low‐intensity fires in this fire‐adapted ecosystem. We found that the lizards adjusted energy allocation away from sexual signaling and growth in response to low‐intensity fires. As fires and fire management are likely to increase in response to changing fire regimes across the globe, it will be important to consider behavioral and physiological responses of impacted species, as well as population‐, community‐, and ecosystem‐level responses.

## Graphical abstract

We show that environmental stress resulting from low‐intensity fire reduced the expression of a condition‐dependent ornament. Female striped plateau lizards had smaller and less orange dewlap ornaments in the month following the Horseshoe 2 Fire in 2011 than 2 years prior, and on the burned site relative to a nearby unburned site even in the absence of detectable differences in stress hormone levels. We also consider the effects of the fire on body size, body condition, and ectoparasitic mite loads.

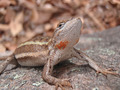

## 1 INTRODUCTION

Wildfire is a powerful ecological and evolutionary factor known to strongly affect ecosystem‐level processes, community structure, plant–pollinator mutualisms, and population dynamics (Banza et al., 2019; Brown et al., 2017; Huang et al., 2016; Pianka & Goodyear, 2012; Smithwick et al., 2005). As fire regimes around the globe change in response to climate change (Abatzoglou & Williams, 2016; Flannigan et al., 2013), it will be important to more fully understand the impacts of wildfire at all ecological levels. It is expected that fires are acute stressors that alter individuals' energy allocation to growth and/or reproduction in important ways. In plants, for instance, wildfire can affect leaf asymmetry (a common measure of plant stress), flowering duration, and nectar concentration (Alves‐Silva & Del‐Claro, 2013; Platt et al., 1988). In animals, it can affect individuals' habitat use, locomotor performance, foraging behavior, growth rate, and more (Brisson et al., 2003; Herzog et al., ,^2014^, ^2016^; Howey et al., 2016; James & M'Closkey, 2003; Wild & Gienger, 2018). Here, we report on an opportunistic study considering the effect of fire on individual phenotype, including physiological stress and the expression of condition‐dependent sexual signals.

**FIGURE 1:**
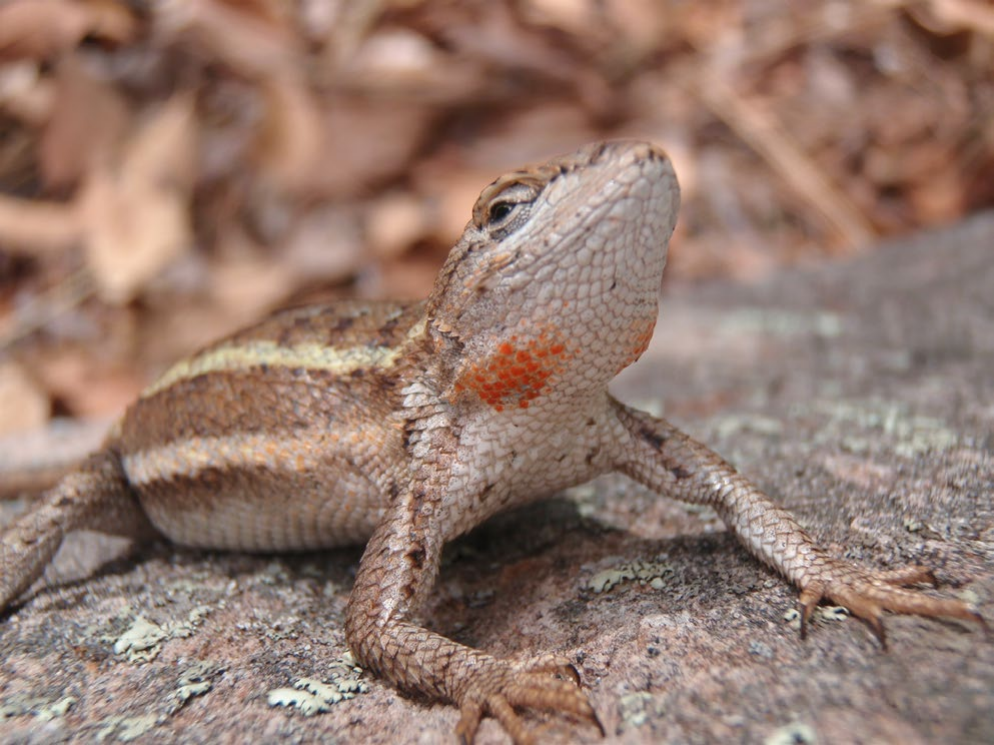
Female striped plateau lizard, *Sceloporus virgatus*

Sexual signals may be sensitive to environmental challenges, such as wildfire, because stress hormones (i.e., glucocorticoids) mediate the energetic and physiological trade‐offs that underlie signal honesty (Buchanan, 2000; French et al., 2007; Husak & Moore, 2008). In brief, elevated glucocorticoid levels trigger the reallocation of energy away from reproduction and regulate the immune system in ways that promote escape, recovery, and survival in the short term, but can be detrimental in the long term. Thus, an individual under chronic physiological stress may have fewer resources to allocate to sexual signals such as song production or ornament expression (Buchanan et al., 2003; Calisi & Hews, 2007; Leary et al., 2006; Roulin et al., 2008; Schull et al., 2018; Spencer et al., 2003). For instance, the experimental elevation of stress hormones led to reduced ornament expression in female striped plateau lizards (*Sceloporus virgatus*, Figure 1; Weiss et al., 2013). The orange throat ornament of *S. virgatus* females develops and fades in synch with the reproductive cycle, peaking around the time of ovulation. Peak color expression is condition‐dependent, varying with female phenotype, as well as the quality of her eggs and offspring (Weiss, 2006, 2016; Weiss et al., 2009), and males modulate their behavior in response to the ornament (Weiss, 2002; Weiss & Dubin, 2018).

*Sceloporus virgatus* is found in the fire‐adapted Madrean Sky Island archipelago in northwestern Mexico, northwest New Mexico, and southeast Arizona. This ecosystem is currently undergoing reorganization in response to climate change and wildfire (Falk, 2013), including a shift from historic regimes of low‐severity, high‐frequency, lightning‐caused fires, to more severe contemporary fires (O'Connor et al., 2014). For instance, the Horseshoe 2 Fire burned more than 70% (222,954 acres) of the Chiricahua Mountain range from 08 May to 25 June 2011, leaving behind a patchwork of areas with varying levels of fire damage from severely burned to unburned (Falk, 2013; Youberg et al., 2013). As part of the fire management efforts, crews conducted burnout operations to remove fuel between the wildfire and a control line. These efforts included burning the land of a long‐term study population of *S. virgatus* in mid‐May, when courtship and female ornament development is typically just beginning (Weiss, 2002). We took advantage of this fortuitous opportunity to examine the effect of this important ecological stressor on female ornament expression. We compared females on the burned site to those on a nearby unburned site (immediately across the control line) in 2011 and 2012, as well as to historical data obtained from females on the burned site in 2009 (preburn). Additionally, we examined female body size, body mass, condition, mite load, and corticosterone, which is the major glucocorticoid hormone in reptiles, birds, and many mammals. If females are sensitive to the ecological stress of fire, we predicted that females on the long‐term study site in the year of the fire (2011) would express reduced ornamentation and other metrics of phenotypic quality relative to (a) females on the unburned site in the same year and (b) females on the same site in a year prior to the fire (2009). We also document the degree of recovery in the year following the fire; we had no a priori prediction as to whether recovery would be so rapid.

## 2 METHODS

Both the long‐term (burned) and unburned study sites are dominated by an oak–pine woodland and follow intermittent streams in Cave Creek drainage, Coronado National Forest, southeastern Arizona, USA. The long‐term site is 515 m long and 1.7 km SW of the Southwestern Research Station (SWRS), the unburned site is 565 m long and 1.9 km NW of SWRS, and the two sites are 2.1 km apart from each other. The width of each site varies with topography and includes the plateau above the creek and the surrounding slopes. In 2011, we determined the percent of the long‐term study site burned by laying transects perpendicular to the creek bed (from the embankment to the outer limit of the site) every 20 m along the creek and continuously scoring the ground or vegetation under the transect tape as burned or unburned. To compare the abiotic conditions of the burned and unburned sites, we placed HOBO data loggers (Onset Computer Corporation, Bourne, MA) on burned and unburned ground on the burned site and on unburned ground on the unburned site for 9 days in 2011, rotating their placement every other day to the sighting location of a randomly selected female. Data were logged every 15 min, and for each 24‐hr period, we determined minimum, average, and maximum temperature and relative humidity. To compare the canopy cover of the two sites in 2011, we took photographs of the canopy in each cardinal direction at randomly selected locations (*n* = 28/site), overlaid a grid of 160 circles on each one to score as open or covered, and used these data to determine average percent cover.

We carefully censused study sites 18–25 June 2011 and 17–23 June 2012. All sighted *S. virgatus* adult females were collected using a loop of fishing line at the end of a retractable fishing pole, immediately bled from the postorbital sinus (in 2011 only), measured as described below, and released to the exact site of capture. Five individuals were measured in both 2011 and 2012; we randomly selected one observation from each female to include in analyses. Measurements include body size (snout‐to‐vent length; SVL), body mass, ectoparasitic mite load (counted under 8× magnification), and reproductive state (determined by palpation). All females (*n* = 122; mean ± *SE* SVL = 63.2 ± 0.3 mm) were gravid except for three small individuals (SVL = 54, 59.5, and 61 mm) that were still vitellogenic. Body condition was calculated as the residuals from a regression of body mass and SVL^3^. In addition, the right throat patch was photographed under full shade using an Olympus C‐5050 ZOOM 5‐megapixel digital camera and was matched to Munsell color chips (Munsell/MacBeth/Kollmorgen Corporation) under full shade and a standardized light source (Pelican Super SabreLite). Ornament size was determined from photographs using Photoshop to automate the selection of orange pixels and ImageJ to determine the area of the selected pixels. A “color score” was calculated from the Munsell data of hue, chroma, and value following the methods of Burley et al., (1992). We made similar measurements on 18–23 June 2009 (preburn), but only to a cohort of females that were part of an ongoing study tracking change in ornament expression with age; these females were at least 3 years old and relatively large (*n* = 17; mean ± *SE* SVL = 67.3 ± 0.2 mm; Weiss, 2016).

**FIGURE 2:**
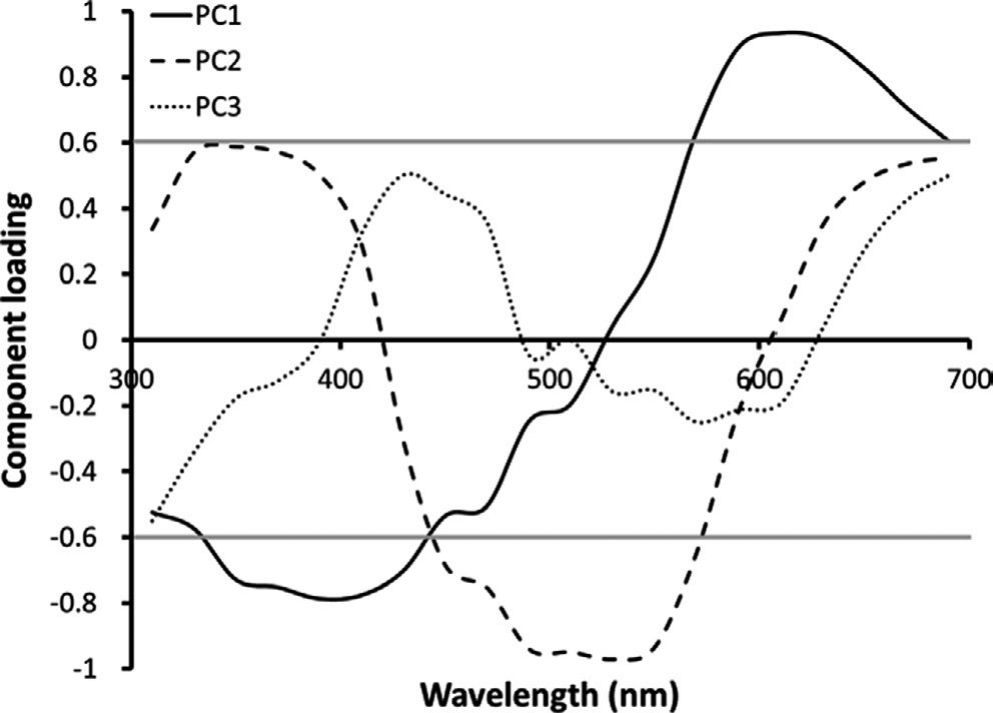
Component loadings across wavelengths from the principal component analysis of ornament color in female *Sceloporus virgatus*. We used a loading of ±0.6 (identified by the gray lines) to define “strong” influences on a given component

In 2011 and 2012, we additionally used an Ocean Optics USB 2000 spectrometer and OOI Base software to quantify ornament color spectra. The ornament was illuminated by a PX‐2 xenon light source, and its reflectance was compared to that of a Spectralon white standard. Spectra were processed as in Weiss et al., (2013). To quantify spectral shape independent from mean reflectance, we subtracted each female's mean reflectance from her spectral data across all wavelengths before submitting the dataset to PCA (Cuthill et al., 1999). Three principal components had eigenvalues >1.0. The first principal component (PC1) summarized 43% of the variance in the spectra; PC1 scores are strongly positively influenced by yellow to red reflectance (>560 nm) and strongly negatively influenced by violet to UV reflectance (<440 nm). PC2 accounted for 38% of the variance; PC2 scores are positively influenced by orange‐red and UV wavelengths and strongly negatively influenced by blue to green reflectance (440–560 nm). PC3 accounted for 9% of the variance; PC3 scores are moderately positively influenced by violet, blue, and red reflectance (420–460 nm and >660 nm) and moderately negatively influenced by extreme UV reflectance (<320 nm). Figure 2 provides the component loadings across wavelengths for each principal component. These three PC scores and mean reflectance were used as response variables in analyses. Mean reflectance indicates the brightness of the ornament, with low reflectance indicating a darker color.

Blood samples were used to compare corticosterone levels of females on the burned and unburned site approximately 1 month after the burnout operation on the long‐term study site. Samples were collected within 4.4 min of an individual being sighted (mean time = 154.4 ± 5.1 s) and were held in a cooler on ice for up to 8 hr before processing. After centrifugation, plasma was frozen for storage and transport to the University of Puget Sound for analysis using a Corticosterone Enzyme Immunoassay Kit (Enzo Life Sciences #ADI‐900‐097) optimized for *S. virgatus* plasma. Samples (burned site: *n* = 25; unburned site: *n* = 23) were run in triplicate with a 1:60 dilution and 2% steroid displacement buffer. Intra‐assay variation was 4.0%, and inter‐assay variation across two assay runs was 3.1%.

### 2.1 Statistical analysis

We compared abiotic conditions between locations (burned site/burned ground, burned site/unburned ground, unburned site/unburned ground) using general linear models, with date as a repeated factor for all variables except canopy cover. We also used general linear models to determine (a) whether female phenotype was affected by site and by year, including 2011 and 2012 only, and (b) whether the phenotype of a subset of large females on the long‐term study site differed before, year of, and 1 y after the fire. The latter analysis included only large females (SVL ≥ 65 mm) because that is all that had been measured in 2009. Body condition was calculated independently for this subset of data, and significant results were followed by pairwise Tukey's HSD tests. For statistical models for the ornamental traits, which are our primary interest, possible covariates included SVL, body condition, and mite load; we used AIC to select which, if any, covariates were included in our final model, and present results for all remaining model covariates.

To meet model assumptions, orange area and mite load were log‐transformed, Munsell scores were transformed to the fourth power, and minimum temperature data were transformed to the tenth power. Analyses conducted on orange area and on relative orange area (i.e., orange area/SVL) led to similar conclusions; only analyses on orange area are presented in detail. All analytical statistics were performed using RStudio (R Core Team, 2020).

## 3 RESULTS

### 3.1 Effect of fire on habitat in 2011

In 2011, we found 136 stretches of burned substrate and 124 stretches of unburned substrate along a total of 1,314.5 m of transects placed on the long‐term study site. On average, the burned stretches (8.03 ± 0.78 m) were significantly longer than the unburned stretches (1.79 ± 0.16m; *F*_1,258_ = 119.59, *p* ≪ 0.001) and we determined that 83% of the substrate was burned by the fire. The burned ground had higher average temperature and lower minimum relative humidity than did nearby unburned ground on the same site, but did not differ from unburned ground on the unburned site (Table 1). In 2011, the burned site had less canopy cover than the unburned site (Table 1), but we do not know the degree to which this pattern was affected by the fire or was pre‐existing.

**TABLE 1:** Abiotic conditions (mean ± *SE*) on the burned and unburned study sites in 2011, the year of the Horseshoe 2 Fire, Coronado National Forest, AZ

Site	Burned		Unburned	
Substrate	Burned	Unburned	Unburned	Test statistic
Min T (°C)	16.4 ± 0.7	16.2 ± 0.4	15.9 ± 0.7	*F*_2,16_ = 2.18
Avg T (°C)	29.1 ± 0.7^a^	27.9 ± 0.5^b^	29.0 ± 0.7^a^	***F*_2,16_ = 5.83** *
Max T (°C)	55.7 ± 2.4^ab^	48.8 ± 1.1^b^	55.8 ± 2.1^a^	***F*_2,16_ = 4.52** *
Min RH	7.0 ± 0.6^a^	9.5 ± 0.6^b^	7.7 ± 0.5^a^	***F*_2,16_ = 6.63** **
Avg RH	21.1 ± 0.8	21.9 ± 1.1	21.9 ± 0.8	*F*_2,16_ = 1.22
Max RH	35.6 ± 1.5	39.0 ± 5.2	43.2 ± 5.9	*F*_2,16_ = 1.92
% Canopy Cover	64.2 ± 3.5^a^		85.2 ± 2.4^b^	***F*_1,54_ = 23.66** ***

Note

Data were tabulated for each 24‐hr period and averaged over 9 days, 21–29 June. T: temperature; RH: relative humidity. Test statistics in bold have *p* < 0.05.

Means with different letters indicate significant pairwise differences.

* *p* < 0.05,

** *p* < 0.01,

*** *p* < 0.001.

### 3.2 Does female phenotype differ on burned and unburned sites?

Females were significantly smaller and lighter on the burned site than on the unburned site in both years (Table 2). Female body condition was influenced by an interaction between site and year (Table 2); in 2011, females on the two sites were in statistically similar condition (*F*_1,47_ = 2.25, *p* = 0.141), but in the subsequent year, females on the burned site tended to be in higher condition than those on the unburned site (*F*_1,72_ = 3.60, *p* = 0.062). Mite load was significantly higher in 2012 than in 2011 on both sites (Table 2). Female plasma corticosterone levels did not differ between the burned and unburned sites approximately 1 month after the burnout operation had occurred on the long‐term study site (*F*_1,46_ = 0.12, *p* = 0.726; Table 2).

**TABLE 2:** Phenotypic characteristics (mean ± *SE*) of *Sceloporus virgatus* females on the burned study site and the unburned study site across years

	Burned site		Unburned site		Test statistics
	2011 (*n* = 24)	2012 (*n* = 40)	2011 (*n* = 25)	2012 (*n* = 34)	
SVL (mm)	62.5 ± 0.6	62.9 ± 0.5	63.8 ± 0.8	64.2 ± 0.6	Year: *F*_1,119_ = 0.27 **Site: *F*_1,119_ = 4.84** * Y × S: *F*_1,119_ < 0.001
Body mass (g)	7.6 ± 0.3	7.9 ± 0.3	8.6 ± 0.0.4	8.3 ± 0.3	Year: *F*_1,119_ = 0.02 **Site: *F*_1,119_ = 4.22** * Y × S: *F*_1,119_ = 0.90
Body condition	−0.05 ± 0.16	0.04 ± 0.10	0.26 ± 0.13	−0.21 ± 0.08	Year: *F*_1,119_ = 2.35 Site: *F*_1,119_ = 0.06 **Y × S: *F*_1,119_ = 5.76** *
Mite load	8.7 ± 1.4	23.6 ± 2.4	11.2 ± 2.2	23.8 ± 2.5	**Year: *F*_1,119_ = 46.64** ^ⱡ^ Site: *F*_1,119_ = 0.53 Y × S: *F*_1,119_ = 0.64
Corticosterone (pg/ml)	198.8 ± 31.3		180.2 ± 15.6		Site: *F*_1,46_ = 0.12

Note

Test statistics in bold have *p* < 0.05.

* *p* < 0.05,

ⱡ *p* ≪ 0.001.

**FIGURE 3:**
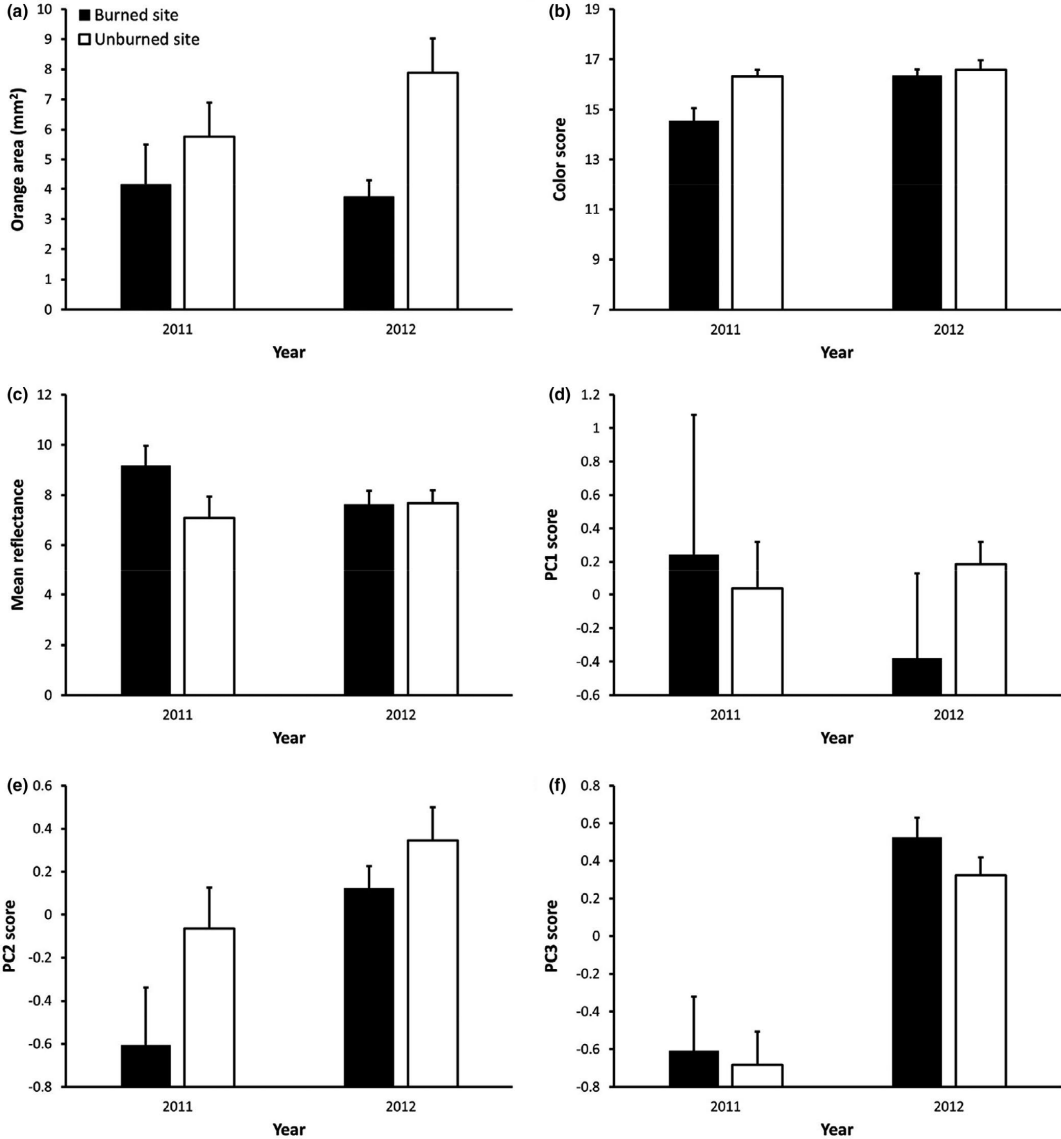
(a) Ornament size and (b–f) coloration of *Sceloporus virgatus* females on the burned site, which burned approximately 1 month prior to measurements in 2011, and a nearby unburned site. The low‐intensity fire was a controlled burn conducted to manage the Horseshoe 2 Fire. (b) Higher color scores represent a darker, more intense orange color. Spectral properties of the ornament were quantified with a spectrometer and PCA resulting in (c) mean reflectance, (d) PC1: relative amount of yellow‐red to UV wavelengths, (e) PC2: relative amount of orange‐red and UV to green‐blue wavelengths, and (f) PC3: high red, blue, and violet to UV wavelengths. Data are means + *SE*

Ornament size and color score both differed between the two study sites (Figure 3a,b). Ornament size was 44% smaller on the burned site than on the unburned site (*F*_1,118_ = 14.33, *p* < 0.001), but was not significantly affected by year (*F*_1,118_ = 1.65, *p* = 0.201) nor a site X year interaction (*F*_1,118_ = 0.02, *p* = 0.884). Female body size was a significant covariate in this model, with larger females having larger ornaments (*F*_1,118_ = 6.30, *p* = 0.013; *β* = 0.03). When relative ornament size was used instead, body size remained in the final statistical model and was marginally significant (*p* = 0.051; *β* = 0.03). Color score was also significantly lower on the burned site relative to the unburned site (*F*_1,119_ = 7.05, *p* = 0.009) and was lower in the year of the fire relative to the subsequent year (*F*_1,119_ = 9.67, *p* = 0.002). The interaction term was not significant (*F*_1,119_ = 2.07, *p* = 0.153), though females on the burned site in the year of the fire tended to have the lowest color score (Figure 3b).

We further assessed four aspects of ornament color using spectrometry (Figure 3c–f): mean reflectance, PC1 (relative amount of yellow‐red to UV wavelengths), PC2 (relative orange‐red and UV to green‐blue wavelengths), and PC3 (relative amount of red, blue, and violet wavelengths to UV wavelengths). Mean reflectance (MR) and PC1 were both unaffected by site (MR: *F*_1,116_ = 1.52, *p* = 0.220; PC1: *F*_1,114_ = 2.55, *p* = 0.113), year (MR: *F*_1,116_ = 0.61, *p* = 0.434; PC1: *F*_1,114_ = 2.37, *p* = 0.126), and their interaction (MR: *F*_1,116_ = 2.34, *p* = 0.129; PC1: *F*_1,114_ = 2.62, *p* = 0.108), though females on the burned site in the year of the fire tended to have the highest mean reflectance. Final models for both reflectance and PC1 included covariates, as follows. Mean reflectance was marginally related to mite load (*F*_1,116_ = 3.51, *p* = 0.063; *β* = −1.90), with heavier mite loads associated with lower reflectance. PC1 was related positively to body size (*F*_1,114_ = 6.61, *p* = 0.011; *β* = 0.06), negatively to body condition (*F*_1,114_ = 7.25, *p* = 0.008; *β* = −0.27), and nonsignificantly to mite load (*F*_1,114_ = 2.44, *p* = 0.121; *β* = −0.43). In contrast, PC2 was lower on the burned site than on the unburned site (*F*_1,116_ = 4.34, *p* = 0.039) and lower in the year of the fire than in the subsequent year (*F*_1,116_ = 11.17, *p* = 0.001) indicating reduced reflection of orange‐red and UV wavelengths in response to stress; the interaction term was not significant (*F*_1,116_ = 0.95, *p* = 0.331), and body size was a significant positive covariate in the model (*F*_1,116_ = 6.52, *p* = 0.012; *β* = 0.062). PC3 was the same on the two study sites (*F*_1,117_ = 0.91, *p* = 0.342), but significantly lower (indicating both reduced red and blue wavelength reflection) in the year of the fire than the following year (*F*_1,117_ = 43.78, *p* ≪ 0.001); there was no significant interaction term (*F*_1,117_ = 0.15, *p* = 0.704) and no remaining covariates.

### 3.3 Does phenotype of the largest subclass of females vary across years relative to the fire?

We compared a subset of the largest one‐third of females (SVL ≥ 65 mm) from the long‐term study site measured in 2011 (year of fire) and 2012 (year after fire), to a cohort of females sampled there in 2009 (preburn) as part of Weiss (2016). Only one individual from that cohort was alive in 2011 and 2012; we randomly selected her 2009 measurement for inclusion in these analyses. Note that only 4 large females are in our 2011 sample, indicative of mortality due to the fire itself. As designed, our subsample did not differ in SVL across years. However, in the year of the fire, large females had significantly lower body mass, body condition, and mite load relative to large females 2 years prior to the fire (Table 3) and in the year subsequent to the fire.

**TABLE 3:** Phenotypic characteristics (mean ± *SE*) of *Sceloporus virgatus* females on the long‐term/burned study site across years

	Long‐term/burned site			
	2009 (*n* = 17)	2011 (*n* = 4)	2012 (*n* = 11)	Test statistic
SVL (mm)	67.3 ± 0.2	67.0 ± 0.7	66.8 ± 0.5	*F*_2,29_ = 0.58
Body mass (g)	10.6 ± 0.2^a^	8.8 ± 0.8^b,^ ^α^	10.0 ± 0.2^a,^ ^α^	***F*_2,29_ = 8.38** **
Body condition	0.40 ± 0.17^a^	−1.34 ± 0.68^b^	−0.12 ± 0.18^a^	***F*_2,29_ = 8.66** **
Mite load	48.3 ± 7.6^a,^ ^α^	5.0 ± 1.6^b^	25.5 ± 5.2^a,^ ^α^	***F*_2,29_ = 14.62** ***

Note

Females from 2011 and 2012 included here are a subset of females with SVL ≥ 65 mm, so SVL is set to be similar across years. Test statistics in bold have *p* < 0.05.

Means with different letters indicate significant pairwise differences.

^α^ Marginal *p* ≤ 0.061,

* *p* ≪ 0.05,

** *p* < 0.01,

*** *p* < 0.001.

**FIGURE 4:**
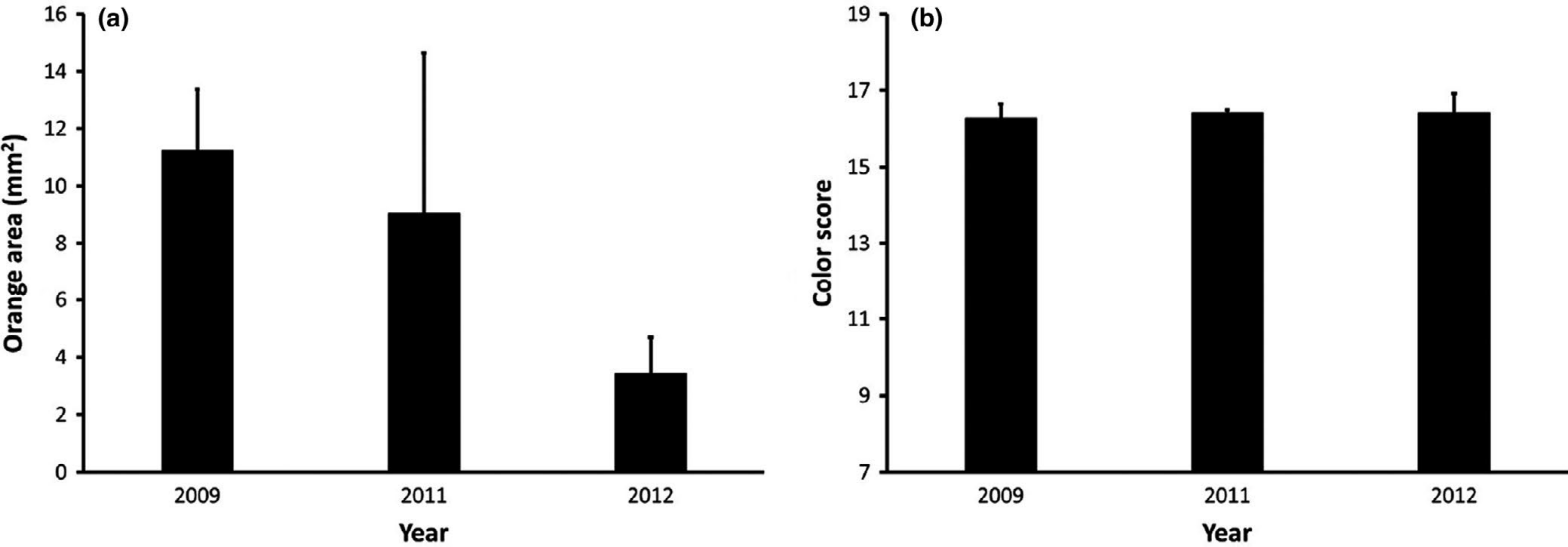
Ornament (a) size and (b) color score of large (SVL ≥ 65 mm) *Sceloporus virgatus* females over years on the Burned site. A controlled low‐intensity fire occurred on the burned site approximately 1 month prior to the 2011 measurements. Data are means + *SE*

Ornament size of large females varied significantly across years (*F*_2,27_ = 9.55, *p* < 0.001; Figure 4a). Relative to large females prior to the fire, the ornaments of large females were ~70% smaller in the year of the fire (*p* = 0.078) and in the subsequent year (*p* < 0.001). There was no difference in ornament size in 2011 and 2012 (*p* = 0.801). Body condition and mite load were both included as covariates in the final model, but neither contributed significantly. Patterns were nearly identical when relative ornament size was used instead of absolute ornament size. Color score of large females did not vary between years (*F*_2,28_ = 0.02, *p* = 0.981; Figure 4b); body size remained as a covariate in the best fit statistical model but did not contribute significantly.

## 4 DISCUSSION

Studies concerning the effect of wildfire and fire management on local fauna typically examine effects on demography, habitat preferences, and thermal tolerances. We believe this is the first to examine the effect of fire on the expression of sexually selected ornamentation. In the year of the Horseshoe 2 Fire, *S. virgatus* females on the burned site developed smaller and less orange ornaments relative to females on the unburned site. Ornament size, but not color, remained suppressed on the burned site in the year after the fire. In previous studies, ornament size has emerged as the stronger signal of female phenotypic quality than has ornament color (reviewed in Weiss & Dubin, 2018), and having a longer period of return to prefire levels may be consistent with that. However, common metrics of quality such as body size and condition showed recovery the year after the fire, whereas ornament size did not, indicating there are additional factors at play that influence annual patterns. Among the largest third of females, ornaments were ~70% smaller in the year of and year after the fire compared with 2 years prior to the fire on the same site, but here, color score was unaffected. Color score is a good indicator of readiness to mate and is closely tied to the ovarian cycle, suggesting that small‐ and average‐sized females were a bit delayed in their reproductive cycle in the year of the fire, while the largest females were not.

The impacts of the fire on ornament expression closely match those from an experimental manipulation of corticosterone in *S. virgatus* (Weiss et al., 2013). In that study, physiological stress was induced in early May (vs. mid‐May in the current study) via long‐term corticosterone implants or tail autotomy. Like in the current study, ornament size and PC2 (relative orange‐red and UV to mid‐wavelengths) were most strongly and negatively affected by the resulting physiological stress. The similar patterns observed in these two studies suggest that the Horseshoe 2 Fire did cause biologically relevant physiological stress, even though females on the burned and unburned study sites did not differ in corticosterone at our time of measurement. It is possible that corticosterone levels surged at the time of the fire and had returned to baseline 1 month later when our sampling occurred. Corticosterone levels of male *Sceloporus undulatus* were also unaffected by low‐intensity fire, when comparing sites 3 months, 6 years, and 50+ years postfire (Iacchetta et al., 2019). Documenting corticosterone levels of wildlife in the days immediately following a fire, and comparing responsiveness among species with different evolutionary histories in fire‐adapted habitats, will be a valuable addition.

Because the sensitivity of the stress response system varies with stressor type, intensity, and duration (Wingfield et al., 1998), we emphasize that both the fires studied here and by Iacchetta et al., (2019) were low‐intensity burns, and our results may not be applicable to populations experiencing high‐intensity fire. Indeed, the lack of a physiological stress response by *S. virgatus* females 1 month following the Horseshoe 2 Fire may be because the habitat was not disturbed strongly enough to generate prolonged thermal challenges to our study population, or these effects were ameliorated by behavioral modifications to habitat use and thermoregulatory behavior (Iacchetta et al., 2019). One month postfire, burned ground was hotter and drier than was unburned ground on the burned site but was similar to unburned ground on the unburned site. Thus, although the fire altered the microhabitat for animals on the long‐term study site, the abiotic conditions were not out of the range experienced by *S. virgatus* in the Chiricahua. We did not measure how the fire impacted the availability of woody debris and other possible refuges and perches. However, in the year after the fire (2012), females on the two study sites did not differ in microhabitat location (e.g., ground, log, rock, or tree) or rate of tail damage, a potential indicator of predation threat (18% on both sites; unpublished data). Stress effects of fire may also have been ameliorated by increased prey availability. Though we did not directly measure prey nor foraging behavior, the *S. virgatus* diet is composed primarily of Coleoptera, Formicidae, and Diptera (Watters, 2008), and fire has been shown to increase the accessibility of some ground‐dwelling insect prey (due to removal of ground cover) and the abundance of fire‐attracted insects (Griffiths & Christian, 1996; Wikars & Schimmel, 2001).

Alternatively, baseline glucocorticoid levels may not be an ideal biomarker of significant environmental change because they are not simply “stress hormones” but also have important roles in every day maintenance of energy balance (Madliger & Love, 2014). Indeed, studies examining the expected positive relationship between glucocorticoids and environmental disturbance show mixed results (reviewed in Madliger et al., 2015). It is possible that glucocorticoids are too labile in response to current external and internal conditions (e.g., weather, time since feeding, recent social interactions, reproductive state) to serve as a reliable metric of disturbance (Madliger et al., 2015). For instance, highly relevant to this study of gravid females, glucocorticoids are often less responsive to environmental stressors during reproduction (Holberton & Wingfield, 2003; Wingfield et al., 1998) and *S. virgatus* females with high corticosterone levels are known to suppress reproduction (Weiss et al., 2013). Perhaps a stronger physiological stress response to fire would be detected outside of the reproductive season.

Body condition, which is often negatively related to baseline glucocorticoid levels (Husak & Moore, 2008), may function as a more stable and integrated phenotypic metric of individual health and fitness consequences of disturbance. On the long‐term study site, body condition of large females was significantly lower in the year of the fire than 2 years prior to the fire, indicating a period of reduced well‐being that is mirrored in the reduction in female ornament expression. This pattern of declining health on the long‐term site is likely due to an interaction between the fire itself and other environmental factors, including a period of extreme drought in 2011 (Weiss, 2016; United States Drought Monitor: droughtmonitor.unl.edu). A comparison between sites could help isolate the effect of the fire itself. In the year of the fire, female body condition was lower on the burned site than on the unburned site; although not statistically significant, the pattern suggests a biologically relevant effect of the fire on fitness‐related traits. Interestingly, in the year following the fire, females on the burned site had little change in condition, whereas females on the unburned site had a large reduction. It is possible that changes in the unburned site reflect continued impact of drought, whereas females on the burned site were buffered from that further reduction. That buffering may have been due to positive changes in habitat structure and/or prey abundances 1 year following the fire (e.g., Griffiths & Christian, 1996). This may suggest that population‐level recovery from low‐intensity fire disturbance can occur relatively quickly and have net long‐term benefits for some herpetofauna.

In conclusion, we examined the effect of low‐intensity fire on female ornament expression, corticosterone levels, body condition, body size, and mite load in *S. virgatus*, a species found within the fire‐adapted Madrean ecosystem. Under this stressor, the lizards shifted their energy allocation away from sexual signaling and growth, and toward basic maintenance, even in the absence of a detectable shift in stress hormone levels 1 month following the acute stressor. These data support the idea that condition‐dependent sexual signals respond to environmental stress (Buchanan, 2000; Husak & Moore, 2008; Weiss et al., 2013) while also challenging the ability of circulating glucocorticoid levels to serve as a biomarker of stress (Madliger & Love, 2014). Interestingly, we found improved body condition the year after the fire, suggesting rapid recovery of *S. virgatus* females to low‐intensity controlled burns. Controlled burns are an important tool to restore native habitat and to manage contemporary fires, which are more severe than historic fire regimes due to global climate change and decades of fire suppression efforts. Of course, herpetofaunal response to fire can vary widely by species (Cano & Leynaud, 2010; Jones et al., 2000; Lindenmayer et al., 2008; McCoy et al., 2013; Pianka & Goodyear, 2012), and impacts are likely dependent upon the health of the habitat prefire and the history of local fire suppression.

## CONFLICT OF INTEREST

The authors have no conflicting interests to report.

## AUTHOR CONTRIBUTIONS

**Stacey L. Weiss:** Conceptualization (lead); Data curation (equal); Formal analysis (lead); Funding acquisition (equal); Investigation (equal); Methodology (equal); Writing‐original draft (lead); Writing‐review & editing (lead). **Robert M. Brower:** Conceptualization (supporting); Data curation (equal); Funding acquisition (equal); Investigation (equal); Methodology (equal).

## Data Availability

Raw data and R scripts are available on the Dryad Digital Repository at https://doi.org/10.5061/dryad.v9s4mw6v3.
